# Stereoselectively fluorinated *N*-heterocycles: a brief survey

**DOI:** 10.3762/bjoc.9.306

**Published:** 2013-11-29

**Authors:** Xiang-Guo Hu, Luke Hunter

**Affiliations:** 1 School of Chemistry, The University of New South Wales, Sydney NSW 2052, Australia

**Keywords:** conformation, fluorine, *N*-heterocycles, iminosugars, medicinal chemistry, organo-fluorine

## Abstract

The stereoselective incorporation of fluorine atoms into *N*-heterocycles can lead to dramatic changes in the molecules’ physical and chemical properties. These changes can be rationally exploited for the benefit of diverse fields such as medicinal chemistry and organocatalysis. This brief review will examine some of the effects that fluorine substitution can have in *N*-heterocycles, including changes to the molecules’ stability, their conformational behaviour, their hydrogen bonding ability, and their basicity. Finally, some methods for the synthesis of stereoselectively fluorinated *N*-heterocycles will also be reviewed.

## Review

### 1. Introduction

A cursory inspection of the medicinal chemistry literature will reveal two obvious themes in the structures of current drug candidates: the ubiquity of nitrogen heterocycles, and the popularity of organofluorine moieties. Therefore, it seems natural that a combination of these two features will offer rich possibilities in the future of drug development. To date, the introduction of fluorine into medicinal entities [1–2] has mostly taken the form of aryl fluorination [3–4] or trifluoromethylation [5–6], and fascinating developments in synthetic methodology of this type are continuing to occur [7–9]. However, with the advent of stereoselective fluorination methods [10–11] it seems clear that the subset of stereoselectively fluorinated *N*-heterocycles [12] offers particularly rich possibilities. We therefore felt that it would be worthwhile to examine in a brief review some of the unique features of this emerging class of molecules.

We have not attempted to cover this topic comprehensively; rather, in the following pages we aim to provide selected examples of the ways that fluorine can influence *N*-heterocycles’ stability and their conformational behaviour; we will see that fluorine can be used as a tool to probe the importance of hydrogen bonding in bioactive molecules; and we will observe how fluorine can affect the basicity of *N*-heterocycles. Finally, we will survey some of the various ways in which stereoselectively fluorinated *N*-heterocycles can be synthesised. Throughout, it will become clear that medicinal chemistry is not the only field that stands to benefit from a deeper understanding of these fascinating molecules: for example, attractive prospects are also clear in the field of organocatalysis [13].

### 2. Fluorination can influence *N*-heterocycles’ stability and reactivity

**Figure 1:**
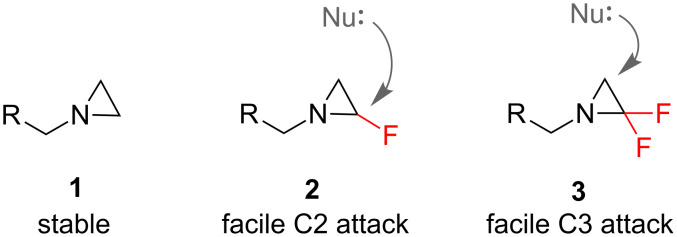
Fluorination alters the reactivity of aziridines.

If a highly-polarised C–F bond is incorporated into a nitrogen heterocycle, it can be expected to have a dramatic influence on the molecules’ physical and chemical properties [14]. The influence that fluorine can have on chemical reactivity is illustrated by considering the smallest *N*-heterocycles, the aziridines. Aziridines (**1**, Figure 1) are generally very stable, in marked contrast with their oxygenated counterparts, the epoxides. However, if one or two fluorine atoms are attached to the aziridine backbone, the resulting molecule is much more susceptible to hydrolysis. De Kimpe and co-workers have investigated the reactivity of mono- and difluoroaziridines **2** and **3** (Figure 1) [15–16]. As well as the enhanced reactivity that **2** and **3** both show towards nucleophilic ring opening, there is an additional subtlety regarding the regioselectivity. While ab initio calculations predict that both **2** and **3** should favour nucleophilic ring opening at C3 [17], preliminary experiments showed that the mono- and difluorinated aziridines actually behave differently in the presence of nucleophiles, with monofluorinated aziridines **2** experiencing C2 attack and the difluorinated counterparts **3** favouring C3 attack.

**Scheme 1:**
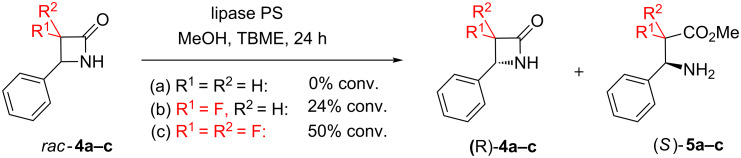
Fluorination makes β-lactam derivatives more reactive towards lipase-catalysed methanolysis.

Fluorination has also been shown to influence reactivity in four-membered *N*-heterocycles (Scheme 1). Kanerva and co-workers [18] investigated a series of β-lactam derivatives (**4a**–**c**) in a lipase-catalysed methanolysis process. While the non-fluorinated derivative **4a** was found to be unreactive under the reaction conditions specified, successive introduction of one or two fluorine atoms (**4b** and **4c**) led to a marked increase in reactivity. The enantioselectivity of this approach is also worthy of note, and will be discussed further in a later section of this review.

Now that our survey of *N*-heterocycles has reached ring sizes of four atoms or larger, another important consideration emerges: fluorine can affect the molecules’ conformational behaviour [19]. To illustrate this point a series of examples are presented below, drawing from heterocycles with ring sizes of up to eight atoms.

### 3. Fluorination can influence the conformations of *N*-heterocycles

#### 3.1 Four-membered rings

**Figure 2:**
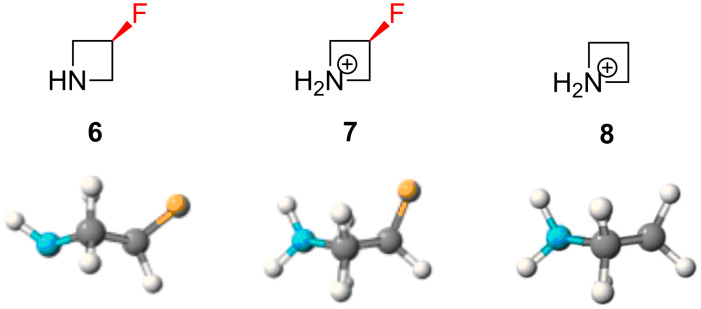
The ring pucker in azetidine derivatives can be influenced by a C–F^…^N^+^ charge–dipole interaction.

O’Hagan and co-workers observed an interesting conformational effect in a computational study of fluorinated azetidine derivatives (Figure 2) [20]. The neutral molecule **6** was calculated to prefer a ring pucker which placed the fluorine atom far away from the neutral nitrogen atom (N–C–C–F dihedral angle = 137.2°). However, the story changed markedly with the charged derivative **7**: in this case, the ring pucker inverted and the fluorine atom more closely approached the charged nitrogen atom (N^+^–C–C–F dihedral angle = 100.0°). This contrast was explained by invoking a favourable interaction between the C–F dipole and the charged N^+^ atom, and the magnitude of this charge–dipole effect is revealed by comparison with the non-fluorinated control molecule **8** in which the ring pucker is less pronounced (N–C–C–H dihedral angle = 102.3°). It transpires that this C–F^…^N^+^ interaction is a general effect which has also been observed in larger *N*-heterocycles, as discussed below.

#### 3.2 Five-membered rings

**Figure 3:**
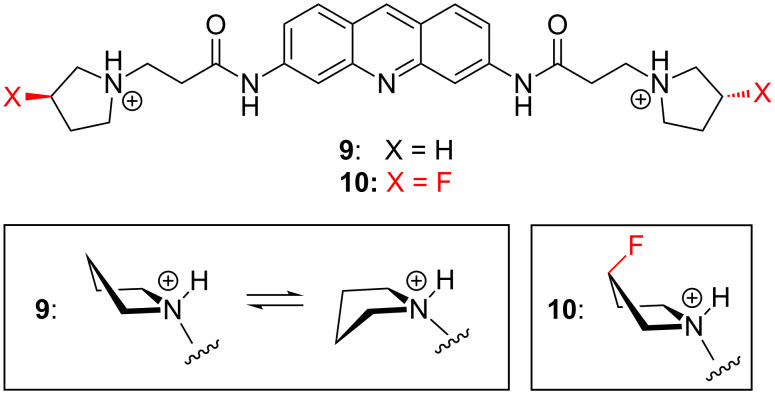
Fluorination ridifies the pyrrolidine rings of ligand **10**, with several consequences for its G-quadruplex DNA binding properties.

The C–F^…^N^+^ interaction can have a more dramatic impact on the conformations of pyrrolidines, since they are inherently more flexible than azetidines [21]. For example, O’Hagan and co-workers investigated the pyrrolidine-containing molecules **9** and **10** (Figure 3) as ligands of G-quadruplex DNA [22]. The non-fluorinated ligand **9** had some conformational disorder because the pyrrolidine rings were able to interconvert between *exo* and *endo* puckers. In contrast, the pyrrolidine rings of fluorinated ligand **10** were more rigid, with the fluorine atoms preferring to occupy an axial position consistent with a favourable C–F^…^N^+^ interaction (worth approximately 5.0 kcal/mol). This led to a number of changes to the DNA binding mode of **10**, including a rotation of the entire pyrrolidine ring by 180° relative to that of **9**, and several different H-bonding contacts with the DNA as a result.

**Figure 4:**
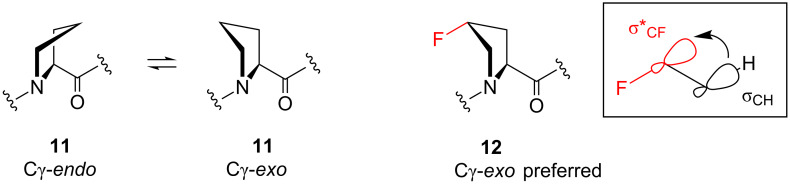
Proline **11** readily undergoes a ring-flip process, but (4*R*)-fluoroproline **12** is more rigid because of hyperconjugation (σ_CH_ → σ*_CF_).

In contrast with the strong charge–dipole effect evident in pyrrolidine **10** (Figure 3), another more subtle interaction is observed in neutral fluorinated pyrrolidines. For example, Raines and co-workers found that (4*R*)-fluoroproline **12** adopts a Cγ-*exo* ring pucker (Figure 4), in contrast with natural proline **11** which has a more flexible pyrrolidine ring [23]. The increased rigidity of **12** was explained by a stabilising hyperconjugation phenomenon (Figure 4), in which an appropriately-aligned σ_CH_ orbital is able to donate electron density into the vacant σ*_CF_ antibonding orbital. This stabilising interaction is only possible if the C–F and C–N bonds are aligned *gauche* to one another, and is analogous to the well-known fluorine *gauche* effect [24]. The importance of the rigid Cγ-*exo* ring pucker of **12** was demonstrated in spectacular fashion: Raines and co-workers showed that the thermal stability of collagen was increased when **12** was incorporated in place of collagen’s naturally-present (4*R*)-hydroxyproline residues [25].

**Scheme 2:**
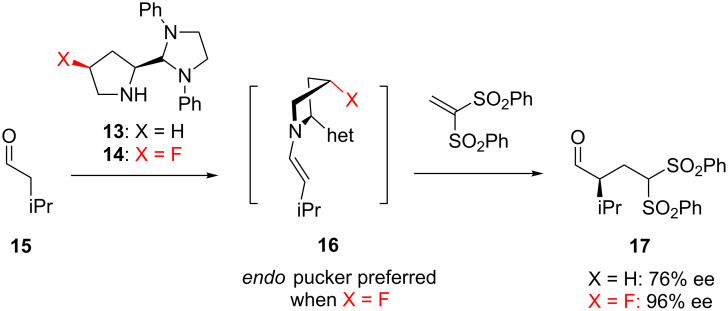
Hyperconjugation rigidifies the ring pucker of a fluorinated organocatalyst **14**, leading to higher enantioselectivity.

This hyperconjugation effect has also been exploited in the context of organocatalysis. Fluorination of proline itself, as well as related *N*-heterocycles, has been shown to increase enantioselectivity in certain organocatalytic processes [13]. For example, Alexakis and co-workers found that the non-fluorinated catalyst **13** (Scheme 2) catalysed an alkylation reaction (**15**→**17**) with only moderate enantioselectivity [26]. This was ascribed to the flexibility of the pyrrolidine moiety in the enamine intermediate **16**. In contrast, the fluorinated catalyst **14** has a relatively strong (1.5 kcal/mol) preference for an *endo* pucker, stabilised by hyperconjugation, and this increased ridigity was credited with a dramatic improvement in the enantioselectivity.

#### 3.3 Six-membered rings

**Figure 5:**
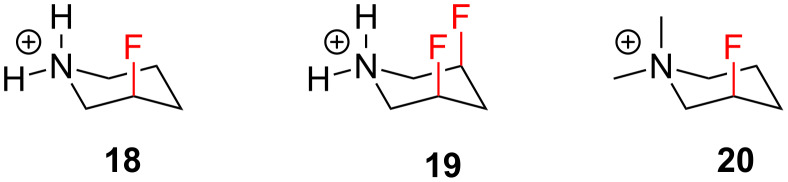
Fluorinated piperidines prefer the axial conformation, due to stabilising C–F^…^N^+^ interactions.

The conformational analysis of six-membered rings is a cornerstone in physical chemistry. Substituted saturated six-membered compounds usually adopt a chair conformation with substituents preferring the equatorial positions. However, in 1993 Lankin and Snyder [27] observed that fluoropiperidine **18** preferentially adopted a conformation in which the fluorine substituent resides in the axial position (Figure 5). This study was then extended to include piperidines **19** and **20**, and in each case the axial conformers are preferred by a substantial ~5.0 kcal/mol over the equatorial conformers (not shown) [28–29]. This pioneering work constituted the original discovery of the C–F^…^N^+^ interaction which has already been discussed above in the context of azetidines and pyrrolidines. Interestingly, Lankin and Snyder were also able to rule out hydrogen bonding as the source of the axial preference, since the *N*,*N*-dimethyl analogue **20** exhibited a similar effect.

#### 3.4 Seven-membered rings

**Figure 6:**
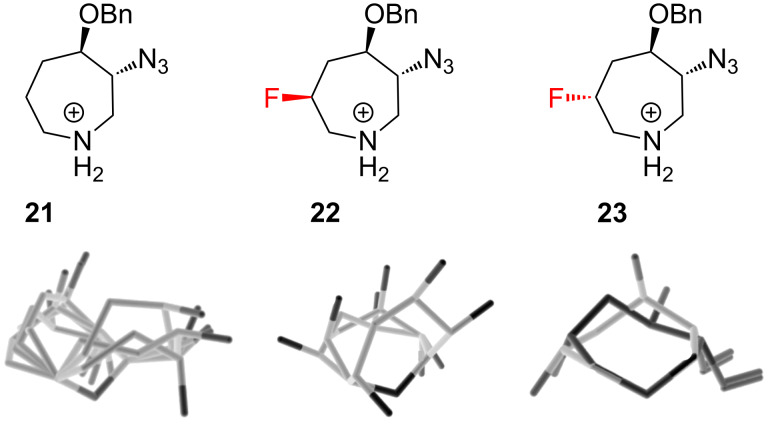
Fluorination can rigidify a substituted azepane, but only if it acts in synergy with the other substituents: azepanes **21** and **22** are disordered, while azepane **23** has one dominant geometry in solution.

Seven-membered rings exhibit much more complex conformational behaviour than six-membered rings. Hence, it is perhaps unsurprising that a twenty year gap separated the pioneering work of Lankin and Snyder (Figure 5) from the first analysis of fluorinated seven-membered *N*-heterocycles. Liu and co-workers [30] have recently explored the conformational behaviour of the substituted azepanes **21**–**23** (Figure 6), and observed that the rigidifying power of a fluorine substituent is strongly dependent on the other groups present. The non-fluorinated azepane **21** was found to exhibit extensive conformational disorder, and this was attributed to competing preferences for the OBn/N_3_ substituents to adopt pseudoequatorial positions and for the azide group to align *gauche* to the ring nitrogen. The situation was not greatly changed upon introduction of a (6*S*)-fluorine atom (compound **22**): in this case, no single conformation of **22** was able to satisfy a C–F^…^N^+^ *gauche* alignment as well as the two conformational preferences described for **21**. In contrast however, introduction of a (6*R*)-fluorine atom (compound **23**) greatly rigidified the ring system, to the extent that a single conformer of **23** dominated in solution. This work highlights the subtleties that can arise when fluorine atoms are incorporated into highly flexible molecules with pre-existing substituents.

#### 3.5 Eight-membered rings

**Figure 7:**
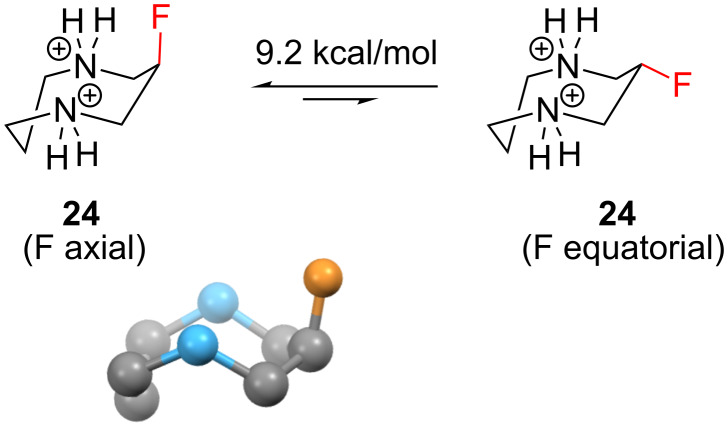
The eight-membered *N*-heterocycle **24** prefers an axial orientation of the fluorine substituent, giving two C–F^…^N^+^ interactions.

The eight-membered ring is the largest stereoselectively fluorinated *N*-heterocycle that has been investigated to date [20]. O’Hagan and co-workers investigated the structure **24** (Figure 7), and calculated that the axial conformation of **24** should be strongly preferred over the equatorial conformation (9.2 kcal/mol) because of two stabilising C–F^…^N^+^ interactions. An X-ray structure of **24** was also obtained (Figure 7), and it revealed a geometry consistent with the calculated minimum-energy structure, with no evidence of disorder.

So far in this review, we have primarily been considering fluorine as a replacement for hydrogen in *N*-heterocycles. However a new vista opens up if we consider fluorine as a replacement for the hydroxy group in bioactive molecules.

### 4. Fluorine can serve as a tool to probe the importance of hydrogen bonding

The replacement of a hydroxy group in a bioactive molecule with a fluorine atom can cause the loss of hydrogen bond donor ability, which may have profound effects on the ligand–receptor interaction. The study of fluorinated iminosugars serves as a good platform to discuss this issue.

**Figure 8:**
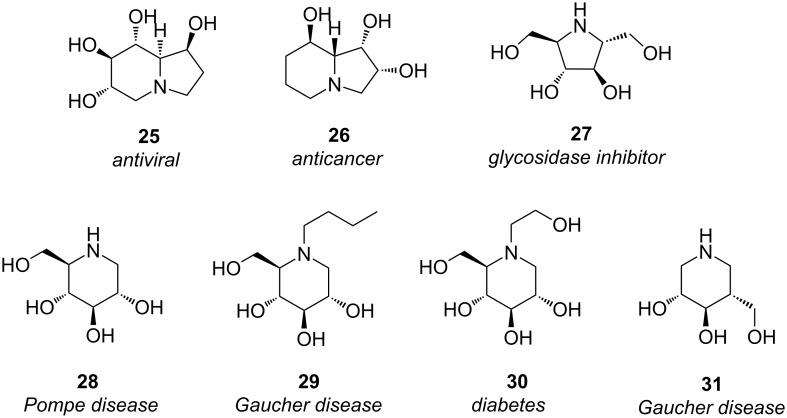
Some iminosugars are “privileged structures” that serve as valuable drug leads.

Naturally occurring iminosugars, also referred as polyhydroxylated alkaloids or azasugars, are sugar mimics in which a nitrogen atom replaces the ring oxygen of the corresponding monosaccharide (Figure 8) [31–36]. Iminosugars can competitively bind to glycosidase enzymes because of their structural resemblance to the terminal sugar moiety of natural substrates, or to the activated intermediate of hydrolysis (i.e. the oxocarbenium ion). As a consequence, iminosugars show great promise for the treatment of a variety of diseases including diabetes, viral infection, bacterial infection, and lysosomal storage disorders [37].

**Figure 9:**
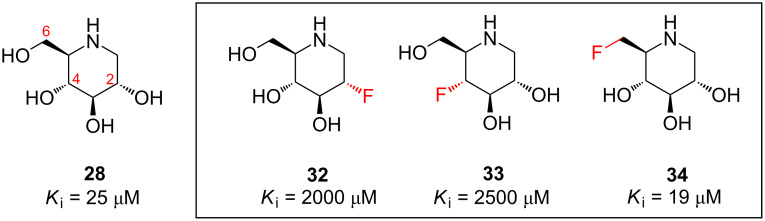
Fluorinated iminosugar analogues **32**–**34** illuminate the binding interactions of the α-glycosidase inhibitor **28**.

Fluorinated analogues of several of these privileged structures have been prepared, in order to probe the importance of hydrogen bonding in these systems [38–43]. For example, 1-deoxynojirimycin (**28**) is the C1-deoxy product of nojirimycin, the first iminosugar isolated from Nature. Iminosugar **28** is a potent inhibitor of yeast α-glycosidase (Figure 9), and the fluorinated analogues **32**–**34** suggest that the C2 and C4 hydroxy groups of **28** act as H-bond donors when binding to the enzyme, while the C6 hydroxy of **28** does not [44–45].

**Figure 10:**
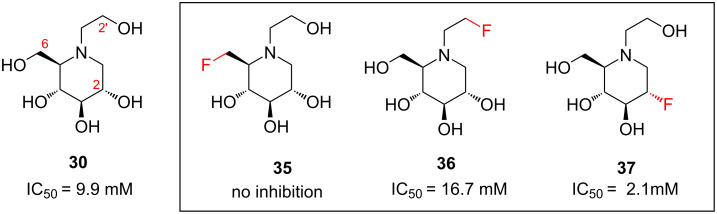
Fluorinated miglitol analogues, and their inhibitory activity towards yeast α-glycosidase.

Miglitol (**30**, Figure 10) is an orally-available drug used for the treatment of type II diabetes. It was first marketed by Merck in 1996. The biological activity of the fluorinated analogues **35**–**37** (Figure 10) suggest that the C6 hydroxy group of **30** acts as a hydrogen bond donor in its binding to yeast α-glycosidase, while the C2' and C2 hydroxy groups of **30** do not [46–47]. The fluorinated analogue **37** is particularly worthy of note, since this compound is five times more potent than the existing drug **30**, and exhibits no toxicity in human cells.

However, a word of warning: in the fluorinated iminosugar examples discussed above (Figure 9 and Figure 10) the inhibition data must be interpreted with some caution, because another effect could be in operation. As well as changing the molecules’ hydrogen bonding properties, fluorination can also affect the basicity of the amine group. This latter effect can be rationally exploited, for example to improve the bioavailability of a drug molecule; this concept is explored in the next section.

### 5. Fluorination alters the basicity of *N*-heterocycles

The 3-piperidinylindole derivative **38** (Table 1) binds to the human 5-HT_2A_ serotonin receptor, and was identified as a promising antipsychotic drug lead [48]. However, the bioavailability of **38** was poor, and this was attributed to the basicity of the secondary amine group which made the molecule positively charged at physiological pH and hence unable to traverse biological membranes. This problem was overcome by introducing a fluorine atom onto the piperidine ring (**39**): the basicity of the secondary amine was thereby reduced by nearly two orders of magnitude, and this led to a marked improvement in bioavailability. Incidentally, it is also worthy of note that the bioavailability (and 5-HT_2A_ binding affinity) could be further improved by the introduction of a second fluorine atom, this time onto the indole moiety (**40**); this further improvement in bioavailability was attributed to blockage of the metabolic degradation of **38** and **39** which commenced with hydroxylation of the indole moiety.

**Table 1:** Fluorination improves the bioavailability of 3-piperidinylindole derivatives **38**–**40** by reducing the basicity of the secondary amine.

	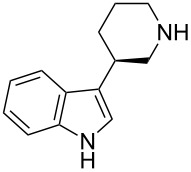 **38**	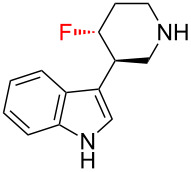 **39**	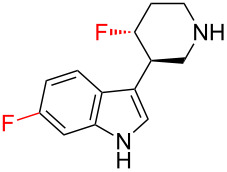 **40**

**p*K*_aH_**	10.4	8.5	~8.5^a^
**5-HT_2A_ affinity**	0.99 nM	0.43 nM	0.06 nM
**Bioavailability**	“Poor”	18%	80%

^a^Not measured, but assumed to be similar to **39**.

**Figure 11:**
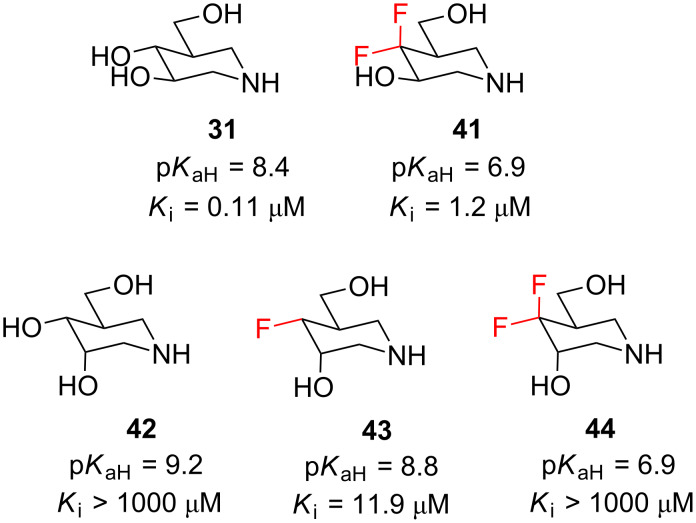
Analogues of isofagomine (**31**) have different p*K*_aH_ values, and therefore exhibit maximal β-glucosidase inhibition at different pH values.

In the next example, we return to the world of iminosugars. Isofagomine (**31**, Figure 11) is an inhibitor of the β-glucosidase from sweet almond, and it is thought to exert its inhibitory activity by mimicking the oxocarbenium intermediate of glycoside cleavage [49]. Several analogues of **31** have been investigated (**41**–**44**, Figure 11) [50–52], and on first inspection it is difficult to rationalise the observed trends in biological activity. One possible explanation for the dramatically improved activity of e.g. **43** over **42** is to invoke the “polar hydrophobic” nature of the fluorine substituent [53–54]. But another important factor is the basicity of the amine group [55]. To best mimic the oxocarbenium ion, the iminosugars **31** and **41**–**44** (Figure 11) must bear a positive charge, and since the p*K*_aH_ values vary considerably amongst the different derivatives, it follows that each derivative has a different “optimal” pH for maximal inhibitory potency. This explains why the *K*_i_ values in Figure 11 do not seem to follow a clear trend: the quoted *K*_i_ values were all measured at the same pH, whereas it would be more revealing to consider the *K*_i_ of each molecule at its “optimal” pH. This is a very interesting situation, because it opens up the possibility of developing drugs that are selective for particular pH environments.

We have now seen that fluorination can affect *N*-heterocycles’ stability, their conformational behaviour, their hydrogen bonding ability, and their basicity. It is hopefully clear to the reader that these effects have already led to several benefits in fields such as medicinal chemistry and organocatalysis. If these concepts are to be continued to be exploited in the future, then robust methods must be available for the synthesis of new fluorinated *N*-heterocycles. Hence, in the final section of this review we will examine some of the stereoselective synthetic methods that have been developed in recent years.

### 6. There are many ways to synthesise stereoselectively fluorinated *N*-heterocycles

#### 6.1 Deoxyfluorination

**Scheme 3:**
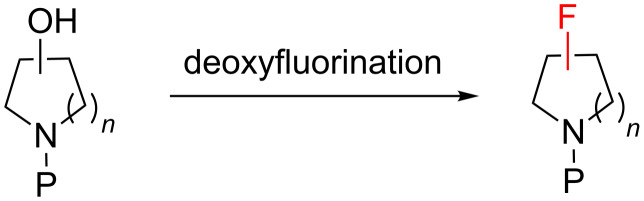
General strategy for the synthesis of fluorinated *N*-heterocycles via deoxyfluorination.

Because of the ease of synthesis of enantiomerically pure alcohols, and the ever-increasing availability of deoxyfluorination reagents [10], the deoxyfluorination of *N*-protected alcohols is the most obvious strategy for synthesising fluorinated *N*-heterocycles (Scheme 3). Deoxyfluorination methods have already been extensively reviewed [8], and of course they are not limited in scope to *N*-heterocyclic systems, so we will not attempt to comprehensively cover this topic here. Instead, we will focus on two recent developments in deoxyfluorination methods that are particularly relevant to *N*-heterocyclic targets.

**Figure 12:**
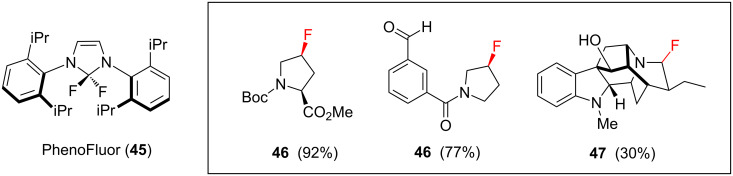
Late stage deoxyfluorination in the synthesis of multifunctional *N*-heterocycles.

Late stage deoxyfluorination is an attractive method for synthesising multifunctional fluorinated *N*-heterocycles, but mild and selective reagents are required if this is to be successfully achieved. One such reagent, PhenoFluor (**45**, Figure 12), was originally developed by Ritter and co-workers for the direct fluorination of phenols [56]. Recent work showed that **45** can also be used to effect late-stage fluorination of hydroxy groups within complex molecular architectures. For example, **45** can react selectively with primary and allylic alcohols in the presence of secondary and tertiary alcohols, and the reaction will also tolerate the presence of carbonyl groups [57]. Some *N*-heterocyclic targets that have been synthesised in one step using **45** as the deoxyfluorination reagent are highlighted in Figure 12.

**Scheme 4:**
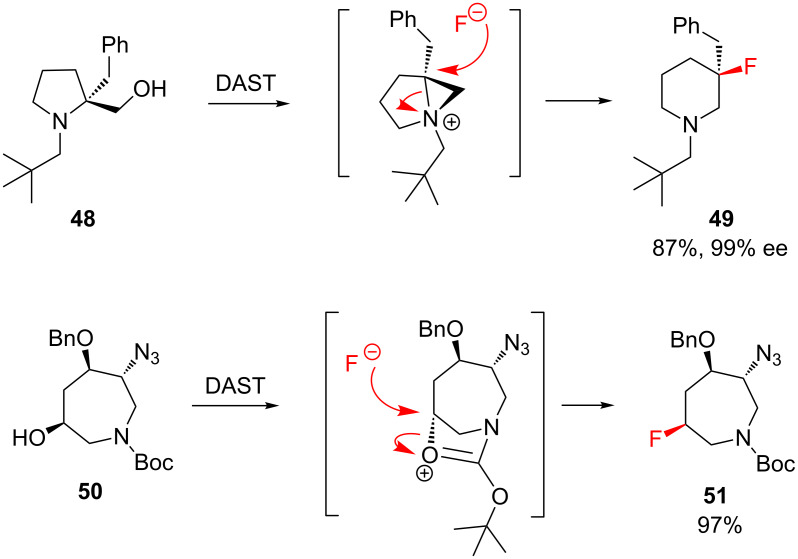
During the deoxyfluorination of *N*-heterocycles, neighbouring group participation can sometimes lead to rearrangement (**48**→**49**) or substitution with retention (**50**→**51**).

A unique complication sometimes arises when deoxyfluorination is attempted in *N*-heterocyclic systems: side reactions can occur, bought about by neighbouring group participation (Scheme 4) [58]. Such processes can lead to rearrangement, and this outcome has been rationally exploited to synthesise fluorinated five- [59], six- [60] and seven-membered [61] *N*-heterocycles that may have been otherwise difficult to access (e.g. **48**→**49**, Scheme 4). Alternatively, neighbouring group participation sometimes results in an unexpected pattern of substitution with retention (e.g. **50**→**51**, Scheme 4); in the latter example, note that the ring nitrogen does not directly engage in the anchimeric process [62].

#### 6.2 The fluorinated building block approach

An alternative to the strategy of deoxyfluorination (section 6.1) is to synthesise fluorinated *N*-heterocycles starting from fluorine-containing organic building blocks. Such an approach benefits from the wide variety, and frequently the low cost, of today’s commercially available organofluorine molecules [63–64].

**Scheme 5:**
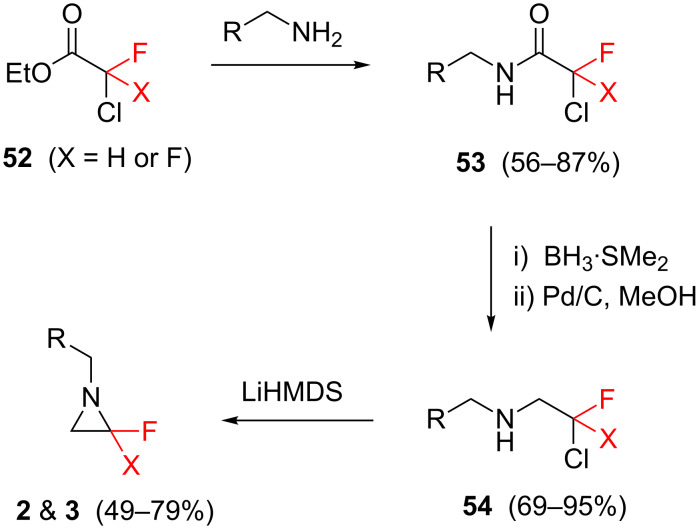
A building block approach for the synthesis of fluorinated aziridines **2** and **3**.

For example, the fluorinated aziridines **2** and **3** presented earlier (Figure 1) were synthesised through a building block approach. De Kimpe and co-workers [15–16] developed a strategy to synthesise such targets via cyclization of β-fluoro-β-chloroamines (**54**, Scheme 5), which in turn are derived from the readily-available fluoroacetate derivatives **52**.

**Scheme 6:**
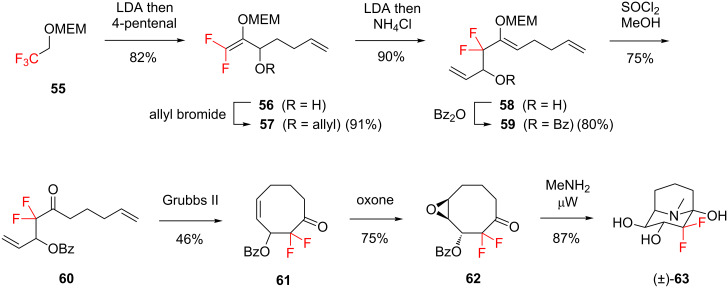
Building block approach for the synthesis of a difluorinated analogue of calystegine B (**63**).

Percy and co-workers’ synthesis of a difluorinated analogue of calystegine B (**63**, Scheme 6) is a more elaborate example of the strategy of using a readily available fluorinated starting material for the synthesis of a complex target [65]. Percy’s approach commenced with protected trifluoroethanol **55** (Scheme 6), and the multistep route to **63** featured a [2,3]-Wittig rearrangement, a diastereoselective epoxidation, and a microwave assisted transannular epoxide opening reaction. It is also noteworthy that the starting material **55** contains an extraneous fluorine atom which is deleted during the synthetic sequence; this approach takes advantage of the often low cost and ready availability of perfluorinated building blocks.

It should be noted, however, that access to enantiopure targets is not straightforward via the building block approach. Such targets may be better obtained through diastereoselective or enantioselective fluorination methods, and examples of these types of approaches are examined in the following sections.

#### 6.3 Diastereoselective fluorocyclisation

**Scheme 7:**
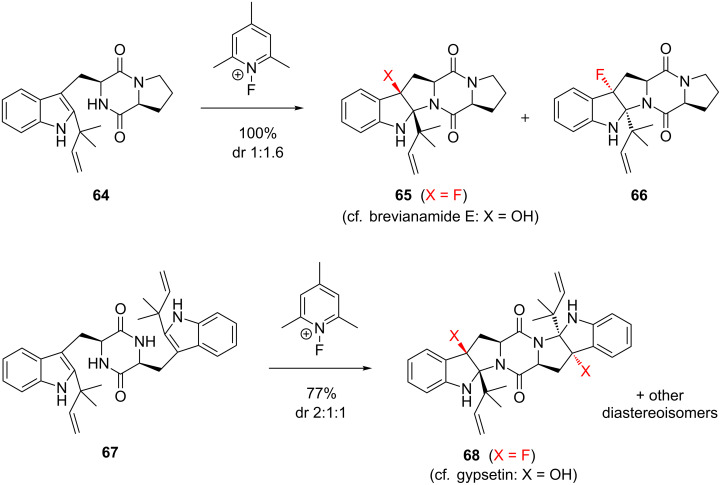
Synthesis of fluorinated analogues of brevianamide E (**65**) and gypsetin (**68**) via electrophilic fluorocyclisation.

The use of fluorocyclisation processes for the production of heterocycles and carbocycles has attracted considerable attention in recent years. Such processes have the advantage of forming multiple bonds in one pot [66]. Electrophilic fluorocyclisation involving the intrinsic nucleophilicity of nitrogen can be a powerful tool to synthesise stereoselectively fluorinated *N*-heterocycles. This concept was exemplified by Shibata and co-workers [67], who in 2001 reported an elegant and efficient method for synthesising fluorinated analogues of the natural product brevianamide E [68] (**65**, Scheme 7). This synthesis was remarkable for its rapid generation of molecular complexity, which is a defining feature of the fluorocyclisation approach. Even more spectacular was the extension of this methodology to create analogues of the natural product gypsetin [69–70] (**68**) via a double fluorocyclisation sequence (Scheme 7). The one drawback of this approach was its disappointing lack of diastereoselectivity, which presumably arose because nonselective fluoroquaternisation of the indole moiety preceded the cyclisation event.

#### 6.4 Enantioselective fluorocyclisation

**Scheme 8:**
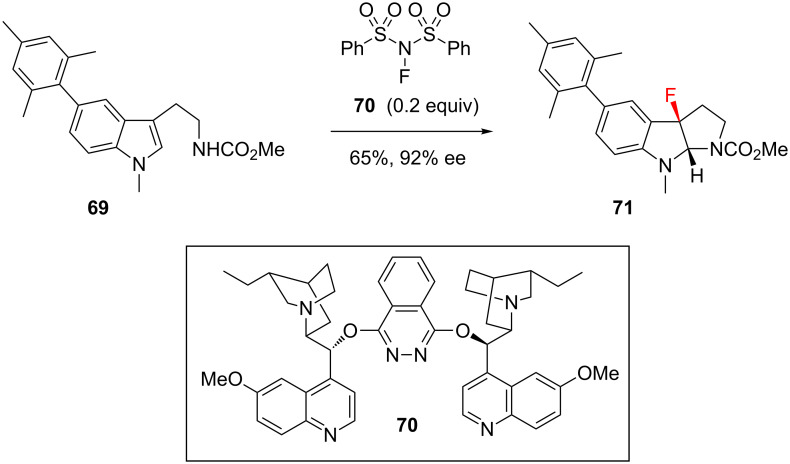
Organocatalysed enantioselective fluorocyclisation.

The lack of diastereoselectivity seen in Scheme 7 is attributable to the fluorination event preceding the cyclisation event, and this is a significant issue which inhibits the further development of diastereoselective processes. However, this issue does not preclude the development of enantioselective variants, provided the initial fluorination event can be controlled [11]. Gouverneur and co-workers recently reported the first enantioselective electrophilic fluorocyclisation (Scheme 8) [71]. Their substrates (e.g. **69**) were indole derivatives bearing a pendant nitrogen nucleophile, and the source of chirality was a substoichiometric quantity of the cinchona alkaloid derivative (DHQ)_2_PHAL (**70**). This method was shown to work very well with several different pendant nucleophiles, but the *N*-acetamido nucleophile was found to be optimal, giving the corresponding product **71** in an impressive 92% ee. Elucidating the mechanism of chiral induction in this type of process is not straightforward, but preliminary experiments showed that associative complexation between the substrate **69** and the alkaloid catalyst **70** may account for the observed enantioselectivity.

#### 6.5 Radical reactions

**Scheme 9:**
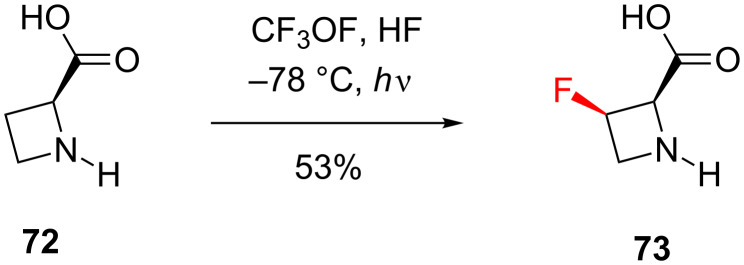
Synthesis of 3-fluoroazetidine **73** via radical fluorination.

Examples of direct fluorination of C–H bonds with fluorine-containing radicals are rare in the literature, especially if stereoselective versions of such reactions are sought. However, this transformation can be a very effective and concise method for synthesising fluorinated *N*-heterocycles. For example, L-*cis*-3-fluoroazetidine-2-carboxylic acid (**73**) was synthesised in one step from the corresponding amino acid **72** by photofluorination with fluoroxytrifluoromethane as the source of the fluorine radical, in 53% yield [72] (Scheme 9).

**Scheme 10:**
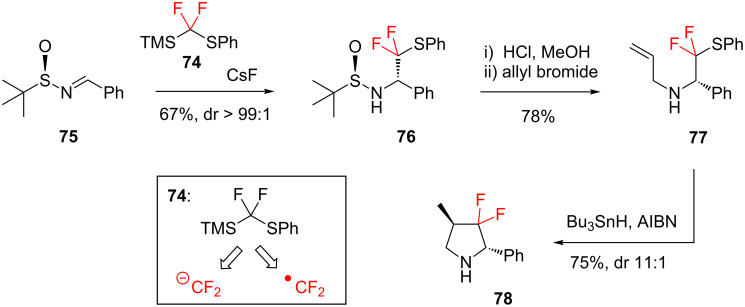
Synthesis of 3,3-difluoropyrrolidine **78** via a radical cyclisation.

Radical reactions can also be used to generate *gem*-difluorinated *N*-heterocycles. For example, Hu and Li [73] employed the versatile reagent **74** (Scheme 10) in their synthesis of the chiral 3,3-difluoropyrrolidine derivative **78**. Reagent **74** can act as either a CF_2_ anion equivalent or a CF_2_ radical equivalent (Scheme 10, inset), and in Hu’s synthesis this reagent fulfils both functions at different stages: thus, the target **78** is achieved from *N*-(*tert*-butylsulfinyl)imine **75** through a nucleophilic addition/radical cyclisation sequence. The selectivity during the radical cyclisation (**77**→**78**) can be explained by the Beckwith–Houk transition-state model [74–75]. The 3,3-difluoropyrrolidine moiety (e.g. **78**) is found in a variety of enzyme inhibitors such as thrombin inhibitors and cathepsin inhibitors, and so this synthetic methodology (Scheme 10) is likely to have valuable future applications in medicinal chemistry.

#### 6.6 Chemoenzymatic synthesis

**Scheme 11:**
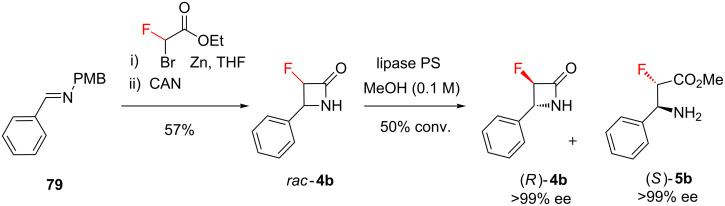
Chemoenzymatic synthesis of fluorinated β-lactam **4b**.

Enzyme catalysis was presented earlier (Scheme 1) as a strategy for synthesising fluorinated β-lactams (**4**) [18]. At that time, we were interested in the effect that the fluorine substituents had on the reactivity of the β-lactam derivatives. However this work now merits further attention, because it also illustrates a strategy for achieving stereoselectivity in C–F bond formation. The racemic β-lactam **4b** was synthesised as a single diastereoisomer from the Schiff base **79** (Scheme 11), by a Reformatsky addition followed by spontaneous cyclisation; removal of the amine protecting group under oxidative conditions then furnished *rac*-**4b**, the substrate for enzymatic resolution. Using an immobilized lipase enzyme as the catalyst (and under slightly different conditions from those described in Scheme 1), one enantiomer of the racemic β-lactam **4b** was completely transformed into the ester **5b**, while the other enantiomer of β-lactam **4b** remained intact. The net result was that a fluorinated stereocentre was rapidly constructed, with defined absolute configuration, within a nitrogen heterocycle.

## Conclusion

When the concept of selective fluorination is applied in the context of *N*-heterocycles, the resulting molecules have a variety of unique properties. In this brief review, we have examined some of these features, including the effects on stability, conformation, hydrogen bonding ability and basicity, and we have also surveyed some of the synthetic methods that are currently available for the production of such molecules. Throughout, we have seen that the unique properties of stereoselectively fluorinated *N*-heterocycles have led to a variety of valuable applications of these molecules, particularly in the field of medicinal chemistry.

What does the future hold? It is interesting to note that the molecules described in this review all comprise ring sizes of three to eight atoms; in contrast, macrocyclic structures have been little explored to date. It will be fascinating to learn whether similar effects operate in much larger ring sizes, for example in fluorinated analogues of cyclic peptides [76–78]. More generally however, it seems safe to predict that the unique properties of stereoselectively fluorinated *N*-heterocycles will ensure that their importance and utility continue to grow in the future.
